# Visualizing Actin Packing and the Effects of Actin Attachment on Lipid Membrane Viscosity Using Molecular Rotors

**DOI:** 10.1021/jacsau.4c00237

**Published:** 2024-04-30

**Authors:** Ion A. Ioannou, Nickolas J. Brooks, Marina K. Kuimova, Yuval Elani

**Affiliations:** † Department of Chemistry, Imperial College London, Molecular Sciences Research Hub, London W12 0BZ, U.K.; ‡ Department of Chemical Engineering, Imperial College London, South Kensington, London SW7 2AZ, U.K.

**Keywords:** actin, vesicles, fluorescence lifetime imaging microscopy (FLIM), membrane packing, molecular rotors, synthetic cell models, membrane biophysics

## Abstract

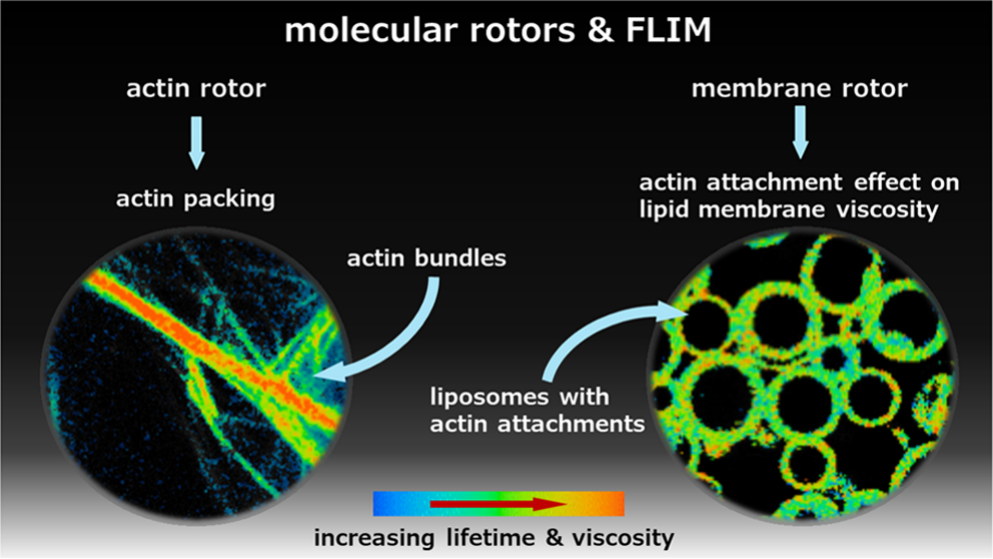

The actin cytoskeleton and its elaborate interplay with the plasma membrane participate in and control numerous biological processes in eukaryotic cells. Malfunction of actin networks and changes in their dynamics are related to various diseases, from actin myopathies to uncontrolled cell growth and tumorigenesis. Importantly, the biophysical and mechanical properties of actin and its assemblies are deeply intertwined with the biological functions of the cytoskeleton. Novel tools to study actin and its associated biophysical features are, therefore, of prime importance. Here we develop a new approach which exploits fluorescence lifetime imaging microscopy (FLIM) and environmentally sensitive fluorophores termed molecular rotors, acting as quantitative microviscosity sensors, to monitor dynamic viscoelastic properties of both actin structures and lipid membranes. In order to reproduce a minimal actin cortex in synthetic cell models, we encapsulated and attached actin networks to the lipid bilayer of giant unilamellar vesicles (GUVs). Using a cyanine-based molecular rotor, DiSC_2_(3), we show that different types of actin bundles are characterized by distinct packing, which can be spatially resolved using FLIM. Similarly, we show that a lipid bilayer-localized molecular rotor can monitor the effects of attaching cross-linked actin networks to the lipid membrane, revealing an increase in membrane viscosity upon actin attachment. Our approach bypasses constraints associated with existing methods for actin imaging, many of which lack the capability for direct visualization of biophysical properties. It can therefore contribute to a deeper understanding of the role that actin plays in fundamental biological processes and help elucidate the underlying biophysics of actin-related diseases.

## Introduction

Actin, being one of the most abundant proteins in eukaryotic cells, has a critical role in a host of cellular processes such as cell movement, division, and structural support.^1^ Actin polymerizes into filaments that constitute the core component of the cytoskeleton, and their function is regulated by actin-binding proteins (ABPs) and various signaling pathways.^2−4^ Dysregulation of actin and ABPs can have extensive implications on cellular function and has been reported to contribute to a wide range of diseases. Among them, actin myopathies can affect the normal function of skeletal muscles and the heart,^5,6^ and abnormalities of the actin cytoskeleton are also associated with cancer development and metastasis. For example, it is well established that overexpression of fascin, an actin-bundling protein, has a crucial role in breast cancer development and can give rise to cancer cell migration, invasion, and metastatic colonization.^7,8^

To date, the most common techniques used to visualize the actin cytoskeleton and ABPs are fluorescence and electron microscopies.^9,10^ The latter can achieve nanometer-range resolution but has limitations when applied to live and wet biological samples due to the high vacuum conditions required in the specimen chamber.^11^ On the contrary, fluorescence microscopy is extensively applied to live cells, and the recent development of super-resolution technologies has enabled researchers to overcome the light diffraction limit and achieve optical resolution down to ∼1 nm.^12−15^ The development of numerous fluorescent probes and their coupling to actin-binding compounds (*e.g*., phalloidin) and antibodies have also permitted simultaneous visualization of multiple targets with high specificity.^16^ Although both electron and light microscopies constitute powerful tools in cytoskeletal studies, current applications are focused on providing morphological and spatial information without being able to directly elucidate any biophysical and chemical aspects of actin and ABP interactions.

In this study, for the first time, we combine fluorescence lifetime imaging microscopy (FLIM) and environmentally sensitive dyes termed “molecular rotors” to directly measure the viscosity and packing of actin bundles. Furthermore, we use a membrane-localized rotor to directly probe the effect of actin attachment on the viscosity of lipid membranes. The method developed here can thus underpin new studies into the biophysical and structural properties of polymerized actin and its interactions with other cellular components like the lipid membrane.

**Figure 1:**
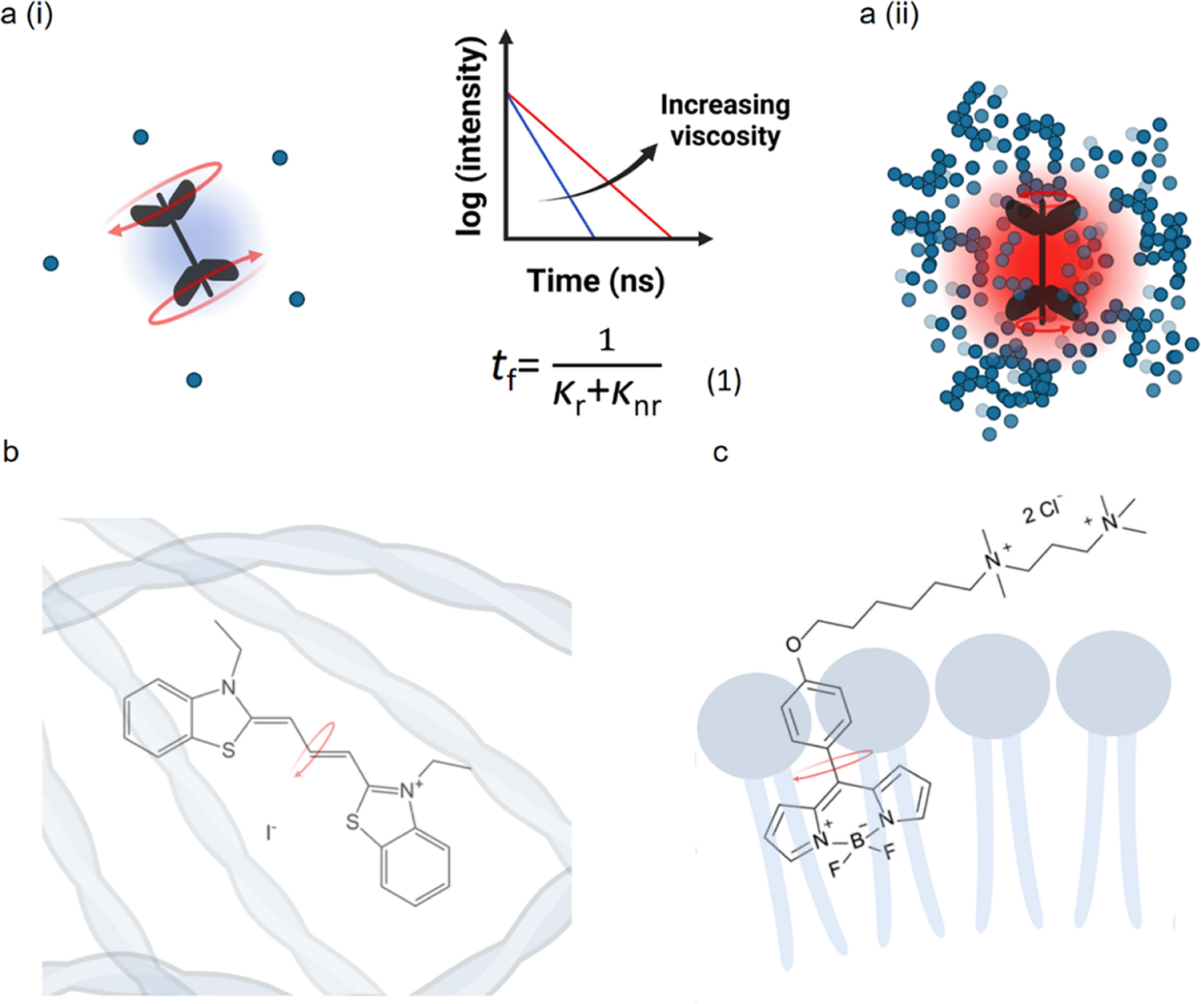
(a (i)) In a less crowded environment (lower viscosity), the intramolecular rotation of a molecular rotor is faster and results in lower fluorescence intensity and lifetime. (a (ii)) In a more crowded environment, due to steric hindrance, the slower intramolecular rotation leads to increased intensity and lifetime values of the rotor. The graph in the middle demonstrates how increasing viscosity affects the time-resolved decay traces of molecular rotors. Blue color corresponds to a lower viscosity environment (lower lifetime), whereas red represents a trace recorded in a more viscous environment (higher lifetime). The equation below (1) describes the relationship of fluorescence lifetime (*t*_f_) with the radiative (κ_r_) and nonradiative (κ_nr_) decay constants, where *k*_nr_ is affected by molecular crowding. (b) Chemical structure of DiSC_2_(3) in an environment of actin assemblies. (c) Chemical structure of BODIPY++ incorporated in a lipid layer. Red arrows indicate the direction of the hypothesized intramolecular rotation.

Molecular rotors are a group of environmentally sensitive synthetic fluorescent molecules that exhibit two competitive relaxation pathways following excitation: radiative decay (leading to fluorescence emission) and intramolecular rotation (leading to nonradiative decay and emission quenching).^17,18^ The nonradiative decay is affected by local steric hindrance, *e.g*., viscosity or crowding, allowing for the emission intensity and lifetime of molecular rotors to serve as microviscosity indicators of their surrounding environment (Figure 1a). Microviscosity is well described by the concept of the solvent free volume: increased concentrations of small solutes result in increased microviscosity (and rotor lifetime values) whereas dilute solutions of large macromolecules do not affect the rotors’ signal.^19^ FLIM enables the visualization of the spatial distribution of fluorescence lifetime values in every pixel of a sample’s image, *via* confocal or multiphoton-excited microscopy.^20−22^ Importantly, in contrast to fluorescence intensity, fluorescence lifetime is independent of the fluorophore concentration.^23^ Thus, molecular rotors in combination with FLIM constitute a powerful microscopical method suitable to accurately measure the viscosity of microscopic samples, such as synthetic and bacterial lipid membranes, protein aggregates, and cellular organelles.^24−27^

Here, we identified a commercially available cyanine rotor, 3,3-diethylthiacarbocyanine iodide, DiSC_2_(3) (Figure 1b), that not only can probe viscosity variations of actin bundles in detail but also can distinguish bundles with different interfilamentous architectures. Following a similar approach, we next used a BODIPY-based rotor (Figure 1c) to monitor lipid viscosity variations upon cross-linking actin assemblies with the lipid membrane of artificial cells. Our method allows, for the first time, to visualize actin packing relevant to structural actin cytoskeletal properties, as well as to monitor downstream mechanical changes of the lipid bilayer following the attachment of polymerizing actin assemblies.

## Results

### DiSC_2_(3) Can Monitor Actin Bundling

**Figure 2:**
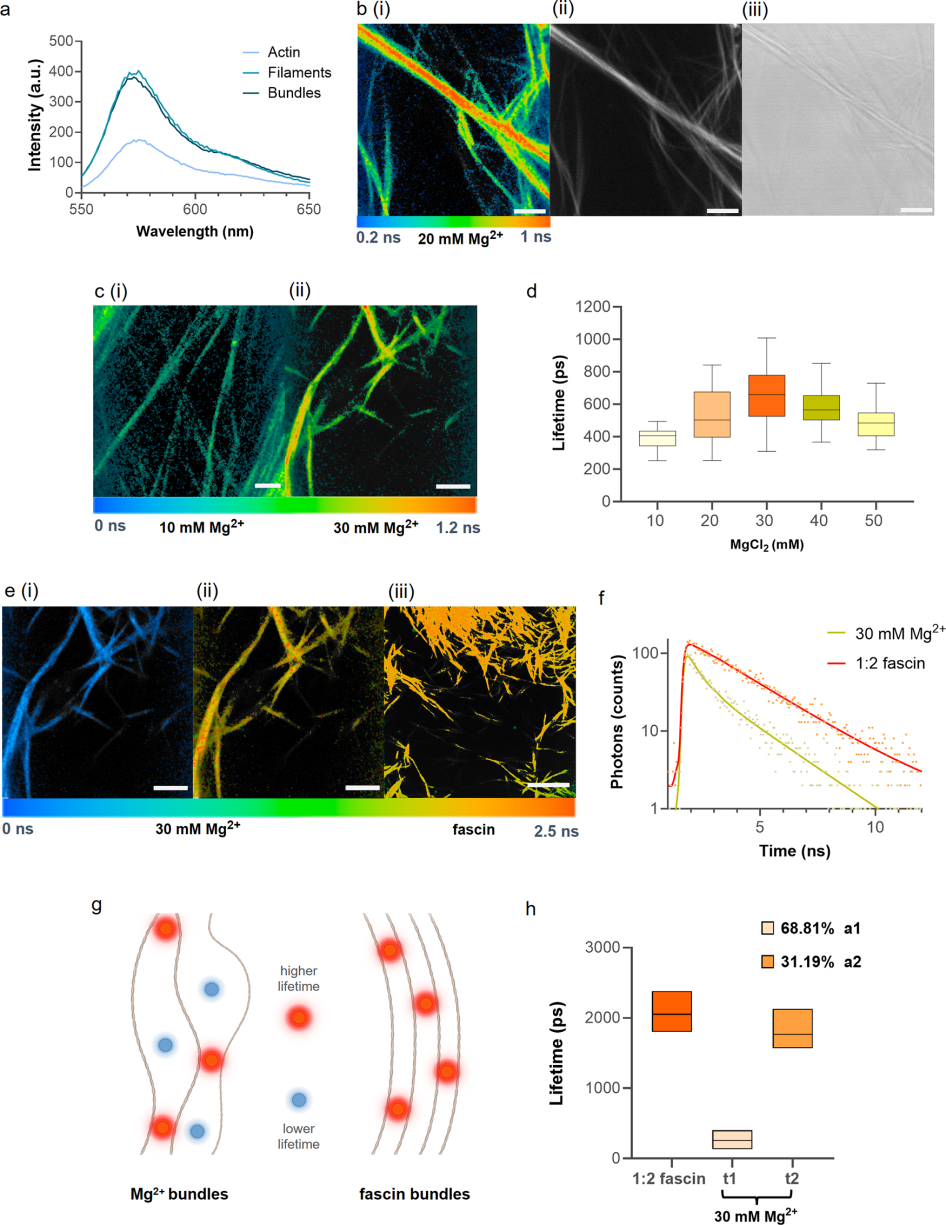
(a) Fluorescence emission spectra of DiSC_2_(3) recorded in the presence of actin monomers, actin filaments, and Mg^2+^-mediated bundles, λ_exc_ 520 nm. (b (i)) FLIM image of 20 mM Mg^2+^-mediated bundles showing regions of higher and lower viscosities. (b (ii)) Fluorescence intensity and (b (iii)) brightfield images of the same sample. (c) FLIM images of 10 mM Mg^2+^(ci) and 30 mM Mg^2+^(cii) bundles; the latter shows higher viscosity. (d) Amplitude-weighted lifetime distribution of bundles formed at varying Mg^2+^ concentrations. Statistical analysis is presented in Figure S4c. Lifetime values from a total of 300 ROIs from FLIM images were analyzed (*n* = 3 independent repeats). (e) FLIM images of t1 (e (i)) and t2 (e (ii)) of Mg^2+^ bundles in comparison with a single lifetime distribution recorded for fascin bundles (e (iii)). (f) Fluorescence decay traces of 30 mM Mg^2+^- and fascin-mediated bundles fitted with monoexponential and biexponential models, respectively. (g) Schematic with the proposed model of interaction of DiSC_2_(3) and the two types of bundles formed. Mg^2+^ bundle decays were fitted best with two components, implying regions with higher and lower viscosities present, whereas fascin bundle decays were fitted monoexponentially, indicating an ordered interfilamentous structure with a single DiSC_2_(3) environment. (h) Comparison of lifetime distribution seen for fascin bundles with distributions seen for Mg^2+^ bundles (*n* = 3). Mean percentage ratios (amplitudes a1 and a2) of t1 and t2 are also shown. Scale bars in (b, c, e (i), and e (ii)) are 5 μm and in (e (iii)) is 10 μm. For FLIM, λ_exc_ 960, λ_emis_ 520–620 nm.

DiSC_2_(3) has previously been shown to act as a hydrophilic molecular rotor with a strong emission intensity and lifetime dependence on the surrounding viscosity, and it has been used to monitor viscosity changes in atmospheric aerosols and to detect multiple steps of amyloid aggregation *via* FLIM.^25,28^ To assess the ability of DiSC_2_(3) to probe actin’s polymerization and bundling, we first obtained fluorescence emission spectra of monomeric and filamentous actin, as well as Mg^2+^-mediated actin bundles, suspended in solution, in quartz cuvettes. Increased fluorescence intensity was recorded for the filamentous and bundled actin samples, suggesting they created a more crowded environment for the added rotor than the monomeric actin state (Figure 2a). To visualize and quantify the viscosity variations within polymerizing actin samples, we next performed FLIM. A typical lifetime image of Mg^2+^-mediated actin bundles is shown in Figure 2bi and reveals the areas of lower and higher viscosities (lifetimes) within each field of view. A direct comparison with the intensity image (Figure 2bii) shows that bundle regions that appear to be thicker present higher lifetimes, suggesting more packed actin assemblies and vice versa. Colocalization of actin rotor DiSC_2_(3) with polymerized actin in Mg^2+^-induced actin bundles was verified by Förster resonance energy transfer (FRET) microscopy in the presence of both DiSC_2_(3) and Acti-stain 670 phalloidin, an actin-targeting peptide that only binds to polymerized actin. DiSC_2_(3) served as an FRET donor and was selectively excited at 488 nm. The signal from 670 phalloidin, serving as an FRET acceptor, cannot be detected, unless the FRET donor and acceptor colocalize within <10 nm. An appreciable signal was collected in the acceptor channel at 680–800 nm (Figure S1a,b), showing the colocalization of DiSC_2_(3) with polymerized actin bundles. When only 670 phalloidin was present and upon excitation at 488 nm, no signal was observed in the detection range of DiSC_2_(3), 520–650 nm (Figure S1c).

We then further explored the sensitivity of DiSC_2_(3) toward actin bundling by comparing FLIM measurements obtained for Mg^2+^- and fascin-mediated bundles. Different concentrations of Mg^2+^ have previously been reported to produce bundles with heterogeneous interfilamentous spacing and varying bending stiffness values, all being less rigid than fascin bundles that are tightly packed and ordered.^29−31^ Thus, we produced bundles in a range of Mg^2+^ concentrations (10–50 mM) as well as fascin-induced bundles with a 2:1 molar ratio of actin to fascin. Lifetime values were selected from individual bundles visible in FLIM (Figure S2a). Each of the bundle samples formed under different Mg^2+^ concentrations produced a range of lifetime values (Figure 2d) corresponding to a mixture of bundles with varying viscosities. While DiSC_2_(3) displays a monoexponential decay in homogeneous solutions (*e.g*., methanol/glycerol),^25^ fluorescence decays collected from Mg^2+^ bundles were best fitted to a biexponential decay model (Figures 2e and S2e–h), suggesting two distinct environments, with lower (t1) and higher (t2) lifetime values of 135.7 and 2479 ps, respectively, corresponding to less and more crowded bundle regions (Figure 2f–h). The presence of multiple components was also verified with phasor analysis (see Figure S7b). Further analysis of the FLIM data for all Mg^2+^ bundles (Figure S4) indicate that the lifetime values t1 and t2 remain constant between different Mg^2+^ concentrations and correspond to viscosities of *ca*. 1 and 500 mPa·s, according to the rotor calibration obtained in sucrose/water solutions,^28^ which was deemed the closest match for the present aqueous-based samples (Figure S5). However, the relative amplitudes of the two components change, with the highest mean value for a1, the faster component, seen for 10 mM Mg^2+^ at 84.82% and the lowest, 68.81%, for 30 mM Mg^2+^. The short lifetime component closely corresponds to the lifetime seen for DiSC_2_(3) in water, thus indicating that some fraction of the dye shows unrestricted rotation within the actin bundles. However, the second fraction characterized by a2 and t2 corresponds to conditions of very high crowding (restricted rotation but not fully bound). Our analysis indicates that increased bundle packing (increased average lifetimes) is a result of the increased ratio of denser to looser regions (a2/a1) within a bundle.

Consequently, of all of the Mg^2+^ concentrations studied, the lowest mean lifetime value was observed for the 10 mM Mg^2+^ sample and the highest for the 30 mM Mg^2+^ sample (Figure 2c,d). This trend is in agreement with the study of Castaneda et al., where TIRF microscopy was used to calculate the bending stiffness of bundles formed under the same range of Mg^2+^ concentrations as employed here,^29^ and they further suggested that the rigidity of these bundles is proportional to the microviscosity of their interfilamentous regions.

Next, using the same approach, we explored the influence of fascin on actin bundling. Fascin is a major actin-bundling protein that regulates cell motility and has recently received attention as a promising prognostic marker of metastatic cancer.^32,33^ In contrast to Mg^2+^-mediated bundles, fascin-induced bundles presented monoexponential decays of actin rotor DiSC_2_(3) with characteristic lifetimes between 1.8 and 2.4 ns, implying a homogeneous environment for the rotor in the bundle interfilamentous space (Figure 2e,g,h).

Indeed, previous structural studies on fascin bundles have revealed a dense and ordered architecture due to the ability of fascin to cross-link actin with a distinct interfilament distance of 8 nm.^34^ These equal interfilament spaces created by fascin bundling of actin (Figure 2g, right) are of a size that is likely to significantly restrict the intramolecular movement of actin rotor DiSC_2_(3), but not to completely confine it, preventing its motion. In order to verify that single lifetimes observed in these experiments were not a result of a strong DiSC_2_(3) binding to actin or fascin, we performed time-correlated single photon counting (TCSPC) measurements of samples containing only actin or fascin that produced multiexponential decays (Figure S8). We then compared fascin bundles with the 30 mM Mg^2+^ bundles that presented the higher lifetimes among all Mg^2+^ concentrations studied. Analysis of the contribution of the two components of 30 mM Mg^2+^ bundles (Figure S3m,n) revealed that the longer lifetime (t2) values were lower than those observed for fascin bundles (Figure 2f,h), corresponding to viscosities of *ca*. 6000 mPa·s, indicating that even in the most packed Mg^2+^ bundles, the rotor senses larger interfilamentous spaces compared to the ones of 1:2 fascin: actin-mediated bundles. These results agree well with previous studies that report the increased rigidity of ABP-induced bundles against bundles whose filaments are not cross-linked.^35,36^

### Encapsulation and Attachment of Actin Networks in GUVs

**Figure 3:**
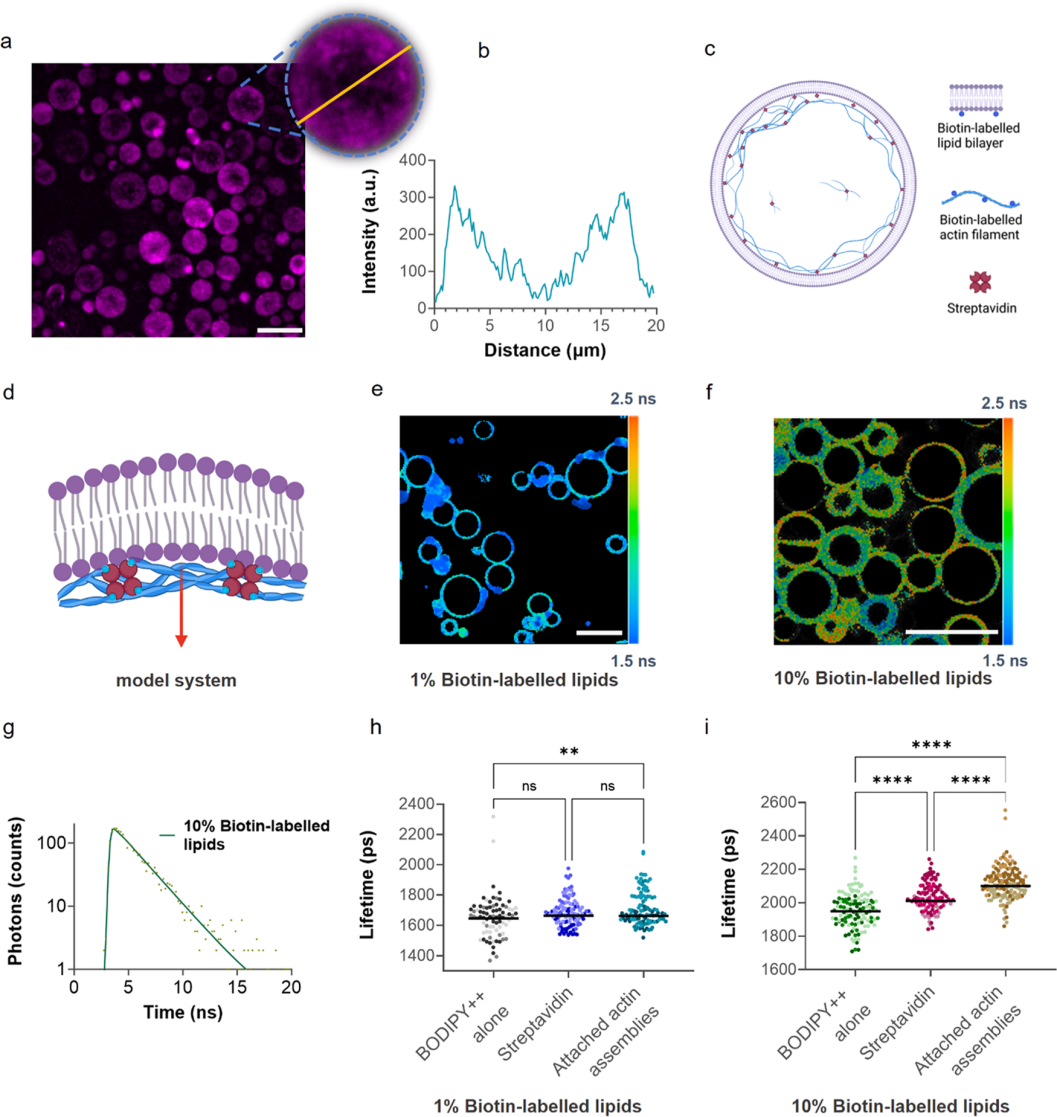
(a) Confocal microscopy image of GUVs (90:10 DOPC:Biotinyl Cap PE) with actin assemblies attached to the inner lipid monolayer. Inset presents a magnified GUV (dashed circle) where actin appears to be localized at the inner periphery of the liposome. 10% mol^–1^ of actin was rhodamine-labeled, 514 nm excitation, 550–650 nm detection. (b) Fluorescence intensity profile of the yellow line across the GUV from inset of (a) where the two peaks of high intensity represent the attachment of actin to the lipid membrane. (c) Schematic presenting the architecture of GUVs with attached actin assemblies and (d) the proposed pulling force (red arrow) of actin assemblies on the lipid layer that should result in a more viscous lipid membrane. (e) FLIM image of 1% biotin-labeled lipid GUVs with attached actin assemblies. (f) FLIM image of 10% biotin-labeled lipid GUVs with attached actin assemblies, 488 nm excitation, 500–600 nm detection. (g) A typical fluorescence decay trace recorded for BODIPY++ incorporated into 10% biotin-labeled lipid GUVs in the presence of actin. Traces were fitted by a monoexponential decay model, consistent with the absence of rotor aggregates in the sample. (h) Lifetime distribution of BODIPY++ in 1% biotin-labeled GUVs, with streptavidin and with attached actin assemblies (376 GUVs analyzed). (i) Lifetime distribution of BODIPY++ with 10% biotin-labeled GUVS (301 GUVs analyzed). All three conditions are significantly different (nonparametric one-way ANOVA, ****: *P* < 0.0001) and GUVs with attached actin assemblies present the highest lifetime values. A total of 677 GUVs were analyzed in three independent repeats, shown as points with different shades of the same color in panels (f, g);. Scale bars at (a, e, f) are 20 μm.

Having demonstrated that FLIM and molecular rotors can probe actin bundling, we then explored whether we can use FLIM to reveal the mechanical effects of actin assemblies on lipid membranes. It is well known that cytoskeleton components can affect the diffusion of species within the lipid bilayers in cells, for example in a “picket and fence” model or when actin attachment can induce lipid phase separation.^37,38^ In our artificial cell model system, we used emulsion phase transfer (EPT) to form GUVs and encapsulate the necessary components for actin networks to form and attach to the inner lipid monolayer. EPT is a well-established method of forming GUVs that offers control of the composition of the encapsulated solution mixture.^39,40^ Aqueous solutions were osmotically balanced with 0.5 M glucose (outer) and 0.5 M maltotriose (inner) in 1× polymerization buffer. We choose to use maltotriose for the inner solution, as its higher density and mass compared to sucrose, which is usually used in EPT, increased the yield of formed liposomes and reduced their movement during FLIM measurements, increasing the signal-to-noise ratio of accumulated data. Networks were formed and attached to the inner lipid monolayer by streptavidin that cross-linked both biotin-labeled actin and biotin-labeled lipids, as was confirmed by fluorescence imaging (Figure 3a). For all experiments, we used a 1:200 ratio of actin to biotin-labeled actin and 2.5 μM streptavidin. Formation of actin networks upon encapsulation was confirmed in a large vesicle population with confocal microscopy, although the distribution from vesicle to vesicle did vary, with some liposomes presenting localization of actin to the periphery of the inner lipid layer and others presenting a homogeneous encapsulation in the bulk (Figure 3b,c).

### Actin Attachment Increases Lipid Packing Density of the Bilayer

We used a bilayer-localized BODIPY++ molecular rotor^41^ (Figure 1c) to investigate the effects of attaching actin networks to the inner lipid monolayer. By combining DOPC and 18:1 Biotinyl Cap PE lipids, we created two conditions at which actin attached to the bilayer: (i) a strong membrane attachment condition in the presence of 10% biotin-labeled lipids and (ii) a weak condition with 1% biotin-labeled lipids. It has been previously reported that streptavidin and avidin attachment to the lipid bilayer increases the membrane rigidity of GUVs.^42^ Thus, for each set of experiments, we also measured two control conditions, one with no proteins added and the other one with streptavidin alone. BODIPY++ is known to localize in the hydrophobic tail region of lipid bilayers, and has previously been extensively used to characterize the packing (microviscosity) of the biological bilayers, whereby monoexponential decays of the rotor can be directly correlated to the viscosity of the fluid lipid phase that it localizes in.^24,41,43^

FLIM analysis was performed on individual GUVs, producing averaged lifetime values for each selected GUV of the FLIM image (Figure S9a). All fluorescence decays fitted well with a monoexponential model (Figures 3g and S9d), indicating good incorporation of the rotor into a single-phase liquid disordered membrane^44^ and absence of dye aggregates that prevent the direct correlation of the rotor’s lifetime with the membrane viscosity.^45^

In weak actin-binding conditions, GUVs with attached actin networks presented the highest lifetime values (Table S1) but no statistical difference between the actin-attached samples and the controls was observed (Figure 3h). However, when the ratio of biotin-labeled lipids was increased to 10% (strong binding conditions), lifetimes of the rotor from GUVs with attached actin were significantly higher than GUVs encapsulating streptavidin or membrane rotor BODIPY++ alone (Figure 3i). While we note that the presence of the higher ratio of biotin-labeled lipids (both with and without streptavidin) also resulted in increased lifetimes compared to the 1% biotin-labeled composition (increase of *ca*. 350 ps and *ca*. 300 ps, respectively), the presence of actin resulted in the biggest change (*ca*. 400 ps further increase). The resulting viscosities that were calculated from the previously determined rotor calibration^28^ are listed in Table S1 and indicate the biggest difference of 98 mPa·s between various conditions tested. These results demonstrate directly how actin assemblies can affect the mechanical properties of lipid membranes.

## Discussion

In recent years, the research of cytoskeletal properties and their association with normal and abnormal cell function has significantly contributed to the fields of cell biology and medicine. In addition to live cell research, *in vitro* studies and the application of synthetic cell models have successfully supplemented this effort.^46−49^ Elucidating the biophysical properties as well as the complex interactions of the cytoskeleton with other cell components such as the plasma membrane will not only reveal key structural and functional aspects of such interactions but also help us to better understand actin-related diseases and cancer. While the use of electron and fluorescence microscopies is inextricably linked with research in the field, it usually provides one-dimensional information (*e.g*., fluorescence intensity-based localization of cytoskeletal components) and can have limitations when applied to *in vivo* samples when using electron microscopies.

Here, by using molecular rotors, we present a FLIM-based approach to study actin packing and the effects of actin attachment to lipid membranes in artificial cells. Dynamic alterations in both environments, actin and lipid bilayers, are relevant in many diseases, and their control could also be important in the design of artificial cells. Uniquely, the packing and microstructure of both environments could be revealed by utilizing our approach of combining environmentally sensitive molecular rotors and FLIM.

We demonstrated how actin rotor DiSC_2_(3) can efficiently monitor and distinguish the microenvironment of two different actin bundle architectures, Mg^2+^- and fascin-mediated bundles, by displaying a wide range of lifetimes (0.25–2.37 ns, corresponding to viscosities of 1–6000 mPa·s), assigned to different bundling conditions and actin morphologies. The recorded wide lifetime ranges for DiSC_2_(3), well below the “saturation” lifetime corresponding to the immobile rotor, also imply a weak interaction between the rotor and actin, enabling its high sensitivity to the crowding in its surrounding environment rather than as a binding conformation probe. The low affinity of DiSC_2_(3) to proteins has also been reported while using FLIM and molecular rotors to monitor amyloid aggregation.^25^

We must note that due to the diffraction-limited resolution of our technique, single filaments of actin could not be resolved with FLIM, but the increased fluorescence intensity of actin rotor DiSC_2_(3) in its presence (Figure 2a) indicates that the rotor is also responsive to that type of actin assemblies. However, the combination of FLIM with super-resolution microscopy, such as stimulated emission depletion (STED)-FLIM, and more sensitive detectors, could be promising for probing finer actin assemblies like thin actin networks and filaments.

By forming and cross-linking a minimal actin cortex to the inner lipid monolayer of GUVs with streptavidin, we used a lipid bilayer-specific molecular rotor, BODIPY++, to investigate potential viscosity variations of the lipid membrane in two attachment conditions. The weak attachment condition (1% biotin-labeled lipids) produced lifetime values with no statistical significance between the controls and GUVs encapsulating actin assemblies. For the strong attachment condition (10% biotin-labeled lipids), membrane rotor BODIPY++ reported higher lifetime values upon actin attachment than GUVs encapsulating the rotor and streptavidin alone, presumably due to actin polymerization and the increased number of adhesion points to the membrane. Generation of membrane tension has previously been proposed in liposomal systems encapsulating actomyosin networks.^47^ However, our results indicate an increased membrane viscosity as a result of actin attachment alone. Indeed, in live cells, the myosin-independent role of actin and adhesion dynamics on membrane tension, especially during cell migration and endocytosis, is well established and has been described in both experimental and computational studies.^50−53^

Lastly, the membrane of GUVs formed with 10% biotin-labeled lipids exhibited significantly higher lifetime values than the 1% composition, even in the absence of actin and streptavidin (Figure 3f,g). We attributed this effect to the increased ratio of 18:1 Biotinyl Cap PE lipids that have bulkier headgroups than DOPC lipids. As previously reported, the presence of lipids with large headgroups (*e.g*., glycolipids, PEG-lipids) can increase the rigidity of model and live cell membranes.^54−56^

We emphasize that this study represents the first instance of using fluorescence lifetime microscopy to measure differences in the biophysical properties of actin assemblies and their effects on lipid membranes. Considering the widespread use of this technique, its sensitivity, and the spatial information it provides, our results represent a significant advancement. This is especially true when coupled with the biomedical significance of actin biophysics and our relatively limited understanding of it, attributable to the deficiencies of existing techniques.

To conclude, we believe that our results pave the way for using FLIM and molecular rotors to monitor actin dynamics and its interactions with lipid membranes. This method can be directly applied to both *in vitro* and *in vivo* cytoskeletal studies of the membrane BODIPY++ rotor. Application of actin rotor DiSC_2_(3) could be extended to experiments in live cells upon attachment to actin-targeting compounds.^57^ Our approach has the potential to provide important insights into both biophysical and morphological aspects of actin and its interactions with lipid membranes, not only in cell structural and motility studies but also in the field of actin-related diseases and cancer.

## Methods

All standard chemicals were purchased from Sigma-Aldrich and were used without further purification. Monomeric, rhodamine and biotin-labeled actin, fascin, and Acti-stain 670 phalloidin were purchased from Cytoskeleton. Streptavidin (recombinant) was purchased from Thermo Scientific. All lipids were purchased from Avanti Polar Lipids: 1,2-dioleoyl-*sn*-glycero-3-phosphocholine (DOPC), 1,2-dioleoyl-*sn*-glycero-3-phosphoethanolamine-*N*-(cap biotinyl) (18:1 Biotinyl Cap PE). Lipid stock solutions were prepared by dissolving lipid powders in chloroform to a concentration of 25 mg mL^–1^ and were stored at −20 °C. BODIPY++ rotor was previously synthesized according to ref (31), and DiSC_2_(3) rotor (99% purity) was purchased from Strateck Scientific.

### Actin Samples Preparation

Monomeric actin was polymerized to a final concentration of 23.4 μM (1 mg mL^–1^) with 10% of 10× polymerization buffer [1 M KCl, 20 mM MgCl_2_, 10 mM EGTA, 0.2 M HEPES–KOH (pH 7.5)] in the presence of 1 mM ATP following standard protocols.^58^ For Mg^2+^-mediated bundles, the actin mixture was supplemented with varying MgCl_2_ concentrations diluted from a 100 mM stock solution in water. DiSC_2_(3) was used at 23.4 μM for all samples as the optimal concentration for the microscopy setup used (Figure S10a). For fascin-induced bundling, 11.7 μM fascin were added to the mixture (2:1 actin: fascin molar ratio) (Figure S10b). For FRET microscopy measurements, Acti-stain 670 was used at 1.2 μM.

### Formation of Giant Unilamellar Vesicles and Attachment of Actin Networks

GUVs were produced with an EPT protocol described by Chiba et al. with slight modifications.^59^ At first, lipid films of 5 mg of lipids were prepared by mixing DOPC and 18:1 Biotinyl Cap PE chloroform solutions in glass phials and dried under a *N*_2_(*g*) flow. Subsequently, lipid films were stored under vacuum for at least 3 h at room temperature before use to ensure total chloroform evaporation. Lipid in oil mixtures were then prepared by dissolving lipid films in mineral oil (5 mg mL^–1^) and sonicated for 60 min at 50 °C. Emulsion droplets were formed by mixing 200 μL of lipid in oil with 20 μL of the solution to be encapsulated inside the GUVs. For control experiments, only maltotriose (0.5 M) and BODIPY++ (0.5 μM) with/without streptavidin (2.5 μM) were encapsulated. For attached actin networks, the inside solution also contained 23.4 μM actin, 0.5% of which was biotin-labeled, 1× polymerization buffer, and 1 mM ATP. The emulsion was then layered above 250 μL of 0.5 M glucose in 1× buffer in 1.5 mL Eppendorf tubes that were centrifuged for 30 min at 10,000 rpm. After centrifugation, the supernatant was removed, and sedimented GUVs were resuspended in 100 μL of 0.5 M glucose. Samples were then transferred in plasma desorption mass spectrometry (PDMS) chambers and incubated for at least 15 min at room temperature to allow for BODIPY++ to incorporate in the lipid bilayer and GUVs to settle before microscopy measurements.

### Fluorescence Lifetime Imaging Microscopy (FLIM)

FLIM measurements were performed in a confocal SP5 II microscope (Leica Microsystems) equipped with a TCSPC card (SPC-830, Becker and Hickl) using either a 63× water immersion objective (NA: 1.2) or a 100× oil immersion (NA:1.4) objective with correction collars. The image resolution was 256 × 256 pixels. Actin samples containing DiSC_2_(3) were excited at 960 nm using a Ti: Sapphire pulsed laser (Chameleon Vision II, Coherent). DiSC_2_(3) emission was recorded between 520 and 650 nm. Liposome samples containing BODIPY++ were excited at 477 nm with a pulsed diode laser (Becker and Hickl GmbH), and emission was collected from 500 to 600 nm. Actin bundle samples (10 μL) were placed on a 24 mm × 50 mm cover glass and sealed with an 18 mm × 18 mm coverslip. For liposome samples, a square 15 mm × 15 mm PDMS chamber was used between the coverslips. SPCImage software (Becker and Hickl) was used to fit fluorescence decays and generate FLIM images. Time-resolved decay traces were fitted using mono- or biexponential decay model. In case of the biexponential decays, average lifetime values were amplitude-weighted and were calculated according to eq 1
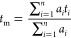
1where a is the amplitude and *t* is the lifetime of the *i*th component. Lifetime values from single bundles and liposomes were extracted using the software’s masking tool (area mode), and graphs were plotted and analyzed with Prism (GraphPad Software).

### Fluorescence Emission Spectra and Lifetime Measurements

Emission spectra were recorded on a Cary Eclipse fluorescence spectrophotometer (Agilent Technologies). DiSC_2_(3) samples were placed in 10 μL, 10 mm path length quartz microcuvettes (Starna Scientific) and excited at 520 nm. Emission spectra were recorded between 550 and 700 nm and corrected for the detector sensitivity. Fluorescence decay traces of DiSC_2_(3) samples were acquired by using a DeltaFlex TCSPC lifetime fluorometer (Horiba Scientific). Excitation was performed with a NanoLED-L 470 nm pulsed laser diode (Horiba Scientific) and detection was set at 570 ± 32 nm. Acquisition was continued until 10,000 peak counts, and the data was analyzed with DAS6 decay analysis software (Horiba Scientific).

## Supporting Information Available

FRET colocalization results; detailed FLIM analysis; full data for lifetime distributions; calibration of molecular rotor lifetimes; phasor analysis data; fluorescence decay traces; data analysis details (PDF)
