# Supplementary material for: Visualizing Actin Packing and the Effects of Actin Attachment on Lipid Membrane Viscosity Using Molecular Rotors
Source: JACS Au. 2024 Apr 30;4(5):2041–9. doi: 10.1021/jacsau.4c00237 (PMC11134356; doi:10.1021/jacsau.4c00237)
Supplement: Supplementary file 1 — au4c00237_si_001.pdf [file au4c00237_si_001.pdf]

Supplementary Information

Visualising actin packing and the effects of actin attachment on lipid membrane viscosity using molecular rotors

Ion A. Ioannou[1,2], Nickolas J. Brooks[1*], Marina Kuimova[1*] and Yuval Elani[2*]

[1] Department of Chemistry, Molecular Sciences Research Hub, Imperial College London, White City, London W12 0BZ, UK

[2] Department of Chemical Engineering, Imperial College London, South Kensington, London, SW7 2AZ, UK

* y.elani@imperial.ac.uk

* m.kuimova@imperial.ac.uk

* n.brooks@imperial.ac.uk

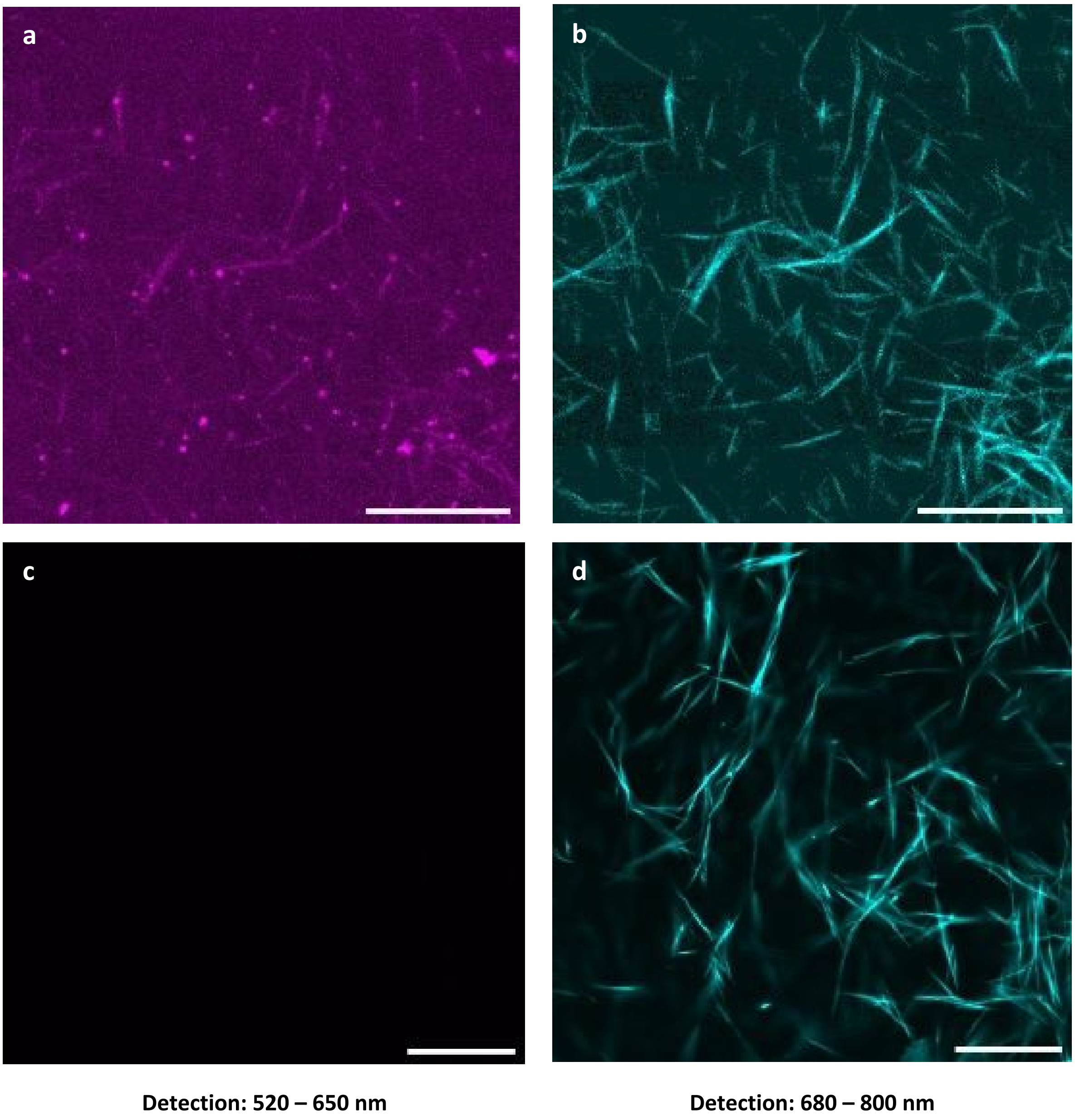

SI Figure 1. FRET microscopy results used for verification of colocalization of DiSC$_2$(3) and Actistain 670 in 50 mM Mg^{2+}-mediated actin bundles. **a)** Fluorescence intensity image of DiSC$_2$(3) serving as FRET donor, upon excitation at 488 nm and detection at 520-650 nm. Bundles appear as thin rods with a weaker intensity signal and a size of ca. 10 µm. **b)** Fluorescence intensity image of Actistain 670 (FRET acceptor) upon excitation at 488 nm and detection at 680-800 nm. **c)** No fluorescence signal can be detected at 520-650 nm (ex:488 nm) where only Actistain 670 is present. **d)** Actin bundles can be clearly distinguished when the same sample as in c is excited at 640 nm and signal from Actistain 670 is collected at 680-800 nm. No signal was observed when Actistain 670 and DiSC2(3) alone were excited at 488 nm with detection at 680- 800 nm. Scalebars are 20 µm. Brighter spots in a) are tentatively assigned to actin aggregates where FRET does not occur since Actistain 670 phalloidin binds only to polymerised actin and not onto aggregates.

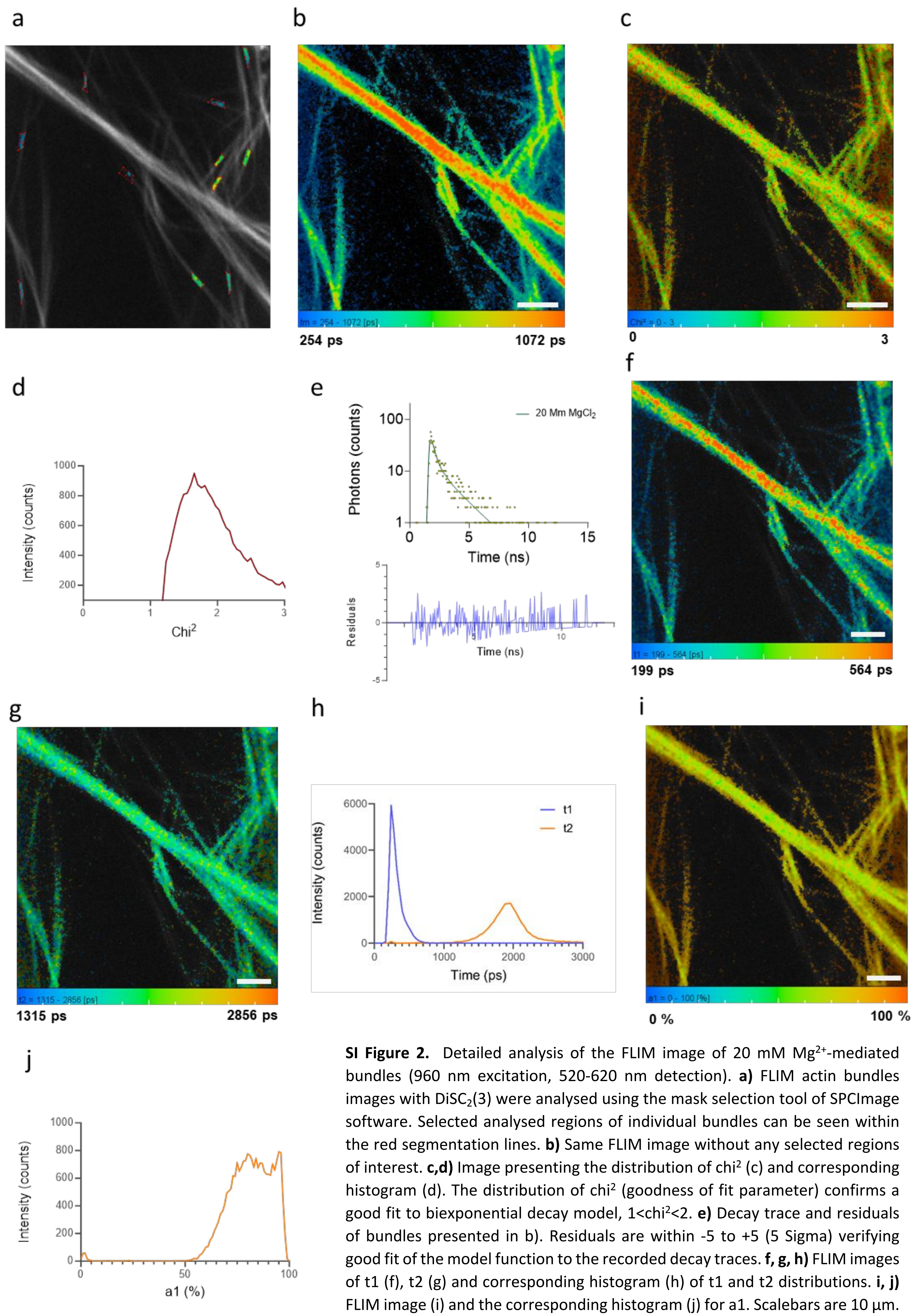

SI Figure 2. Detailed analysis of the FLIM image of 20 mM Mg^{2+}-mediated bundles (960 nm excitation, 520-620 nm detection). **a)** FLIM actin bundles images with $DiSC_2(3)$ were analysed using the mask selection tool of SPCImage software. Selected analysed regions of individual bundles can be seen within the red segmentation lines. **b)** Same FLIM image without any selected regions of interest. **c,d)** Image presenting the distribution of chi^2 (c) and corresponding histogram (d). The distribution of chi^2 (goodness of fit parameter) confirms a good fit to biexponential decay model, 1<chi^2<2. **e)** Decay trace and residuals of bundles presented in b). Residuals are within -5 to +5 (5 Sigma) verifying good fit of the model function to the recorded decay traces. **f, g, h)** FLIM images of t1 (f), t2 (g) and corresponding histogram (h) of t1 and t2 distributions. **i, j)** FLIM image (i) and the corresponding histogram (j) for a1. Scalebars are 10 µm.

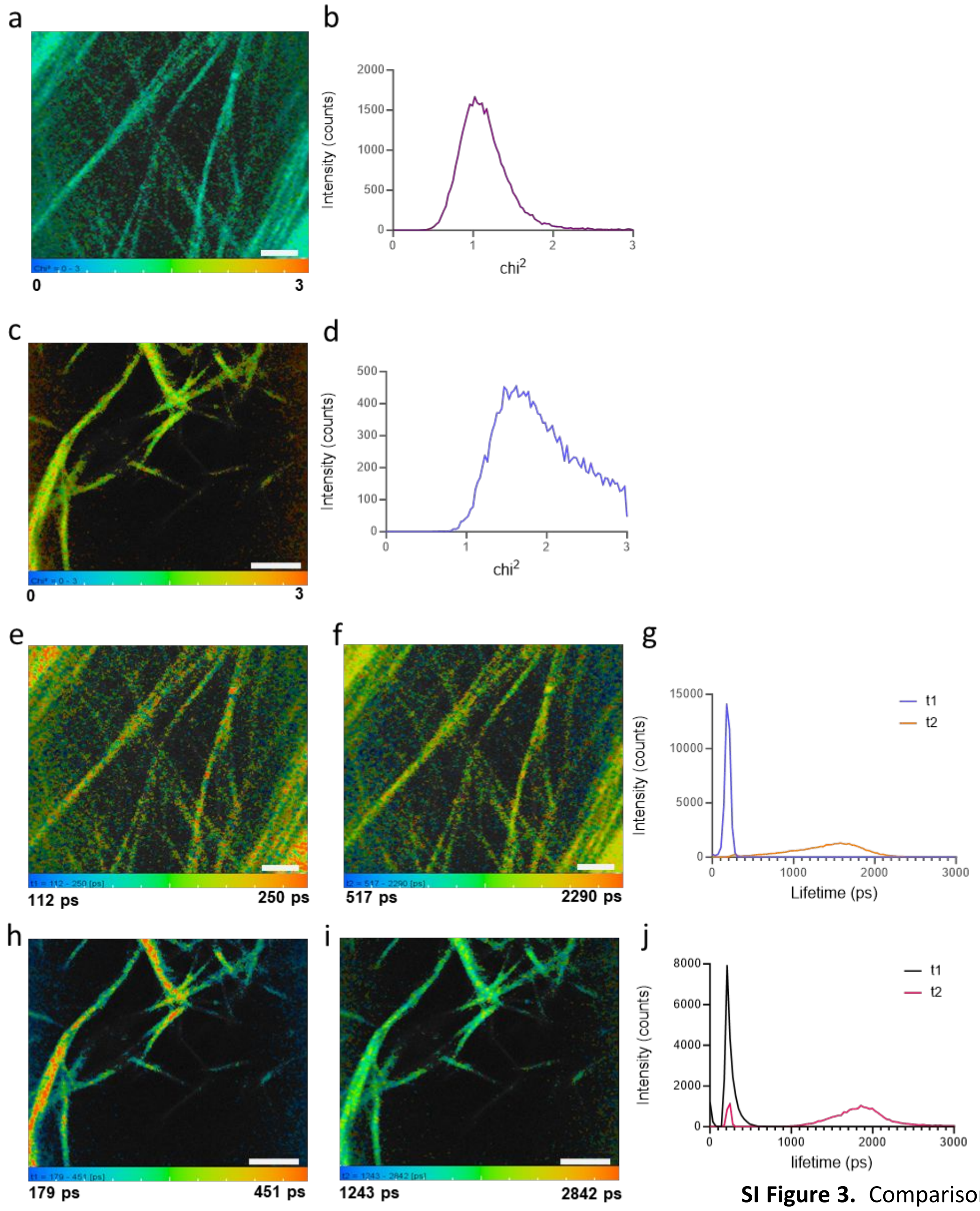

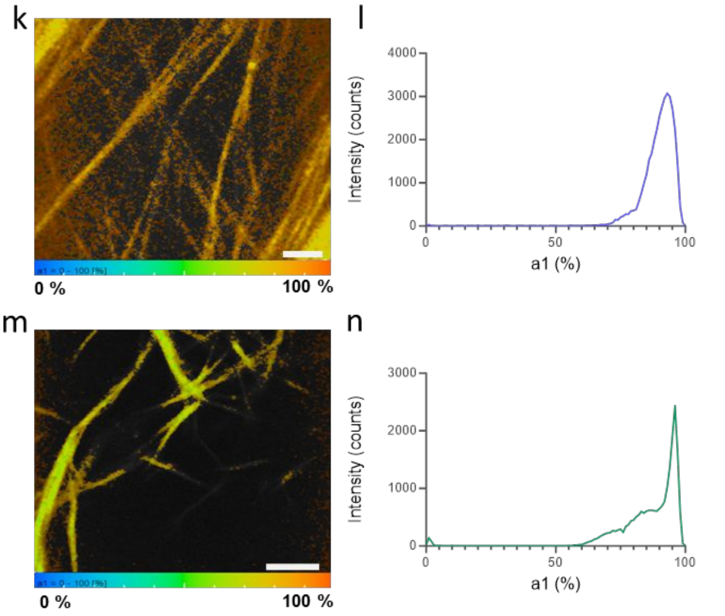

SI Figure 3. Comparison of the FLIM images of 10 and 30 mM Mg^{2+}-mediated bundles of Figure 2c i, ii. **a, b)** Chi^2 distribution image (a) and histogram (b) of 10 mM Mg^{2+}-mediated bundles confirming good fit to the biexponential model. **c, d)** Chi^2 distribution image (c) and histogram (d) of 30 mM Mg^{2+}-mediated bundles **e, f, g)** 10 mM Mg^{2+}-mediated bundles FLIM images of t1 (e), t2 (f) and corresponding histogram (g) of t1 and t2 distributions. **h, i, j)** 30 mM Mg^{2+}-mediated bundles FLIM images of t1 (h), t2 (i) and corresponding histogram (j) of t1 and t2 distributions. **k, l)** FLIM image (k) and the corresponding histogram (l) for a1 of 10 mM Mg^{2+}-mediated bundles. **m, n)** FLIM image (m) and the corresponding histogram (n) for a1 of 30 mM Mg^{2+}-mediated bundles. All scalebars are 5 µm.

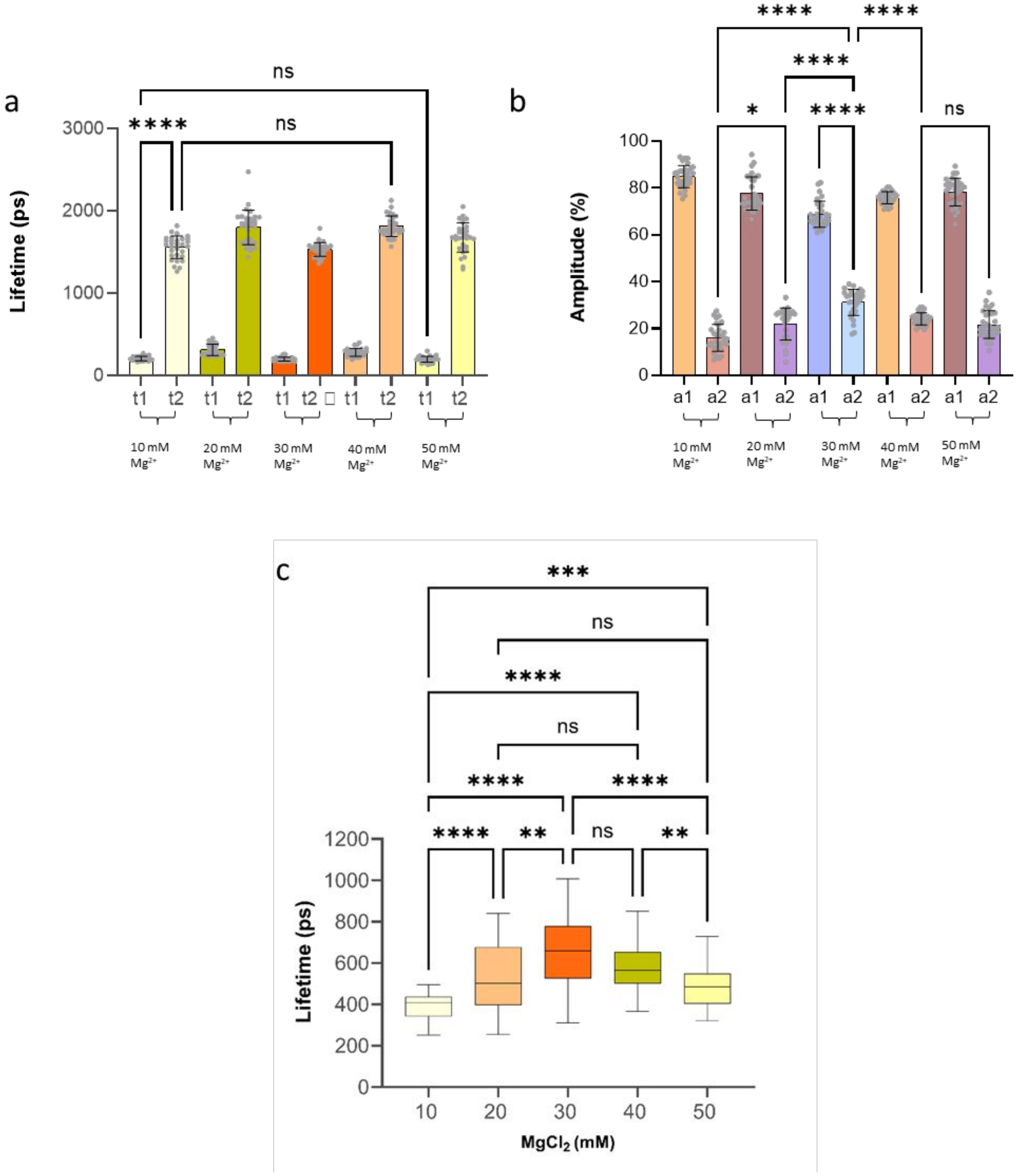

SI Figure 4. a) t1 and t2 distribution histograms of 10 - 50 mM Mg^{2+} bundles. Comparison between values with the smallest mean difference indicated no significant difference amongst t1 values and amongst t2 values. Significant difference is observed between t1 and t2 values. b) a1 and a2 distribution histograms of the same range of Mg^{2+}-mediated bundles. Significant difference is observed between a1 and a2 values. Also, significant difference is observed between a2 values of 10/30 mM Mg^{2+}, 20/30 mM Mg^{2+} and 30/40 mM Mg^{2+} c) Amplitude weighted lifetime distribution of bundles formed at varying Mg^{2+} concentrations from Figure 2d. (One-Way ANOVA, ****: P<0.0001). Lifetime and amplitude values were selected from a total of 150 bundles (n=3).

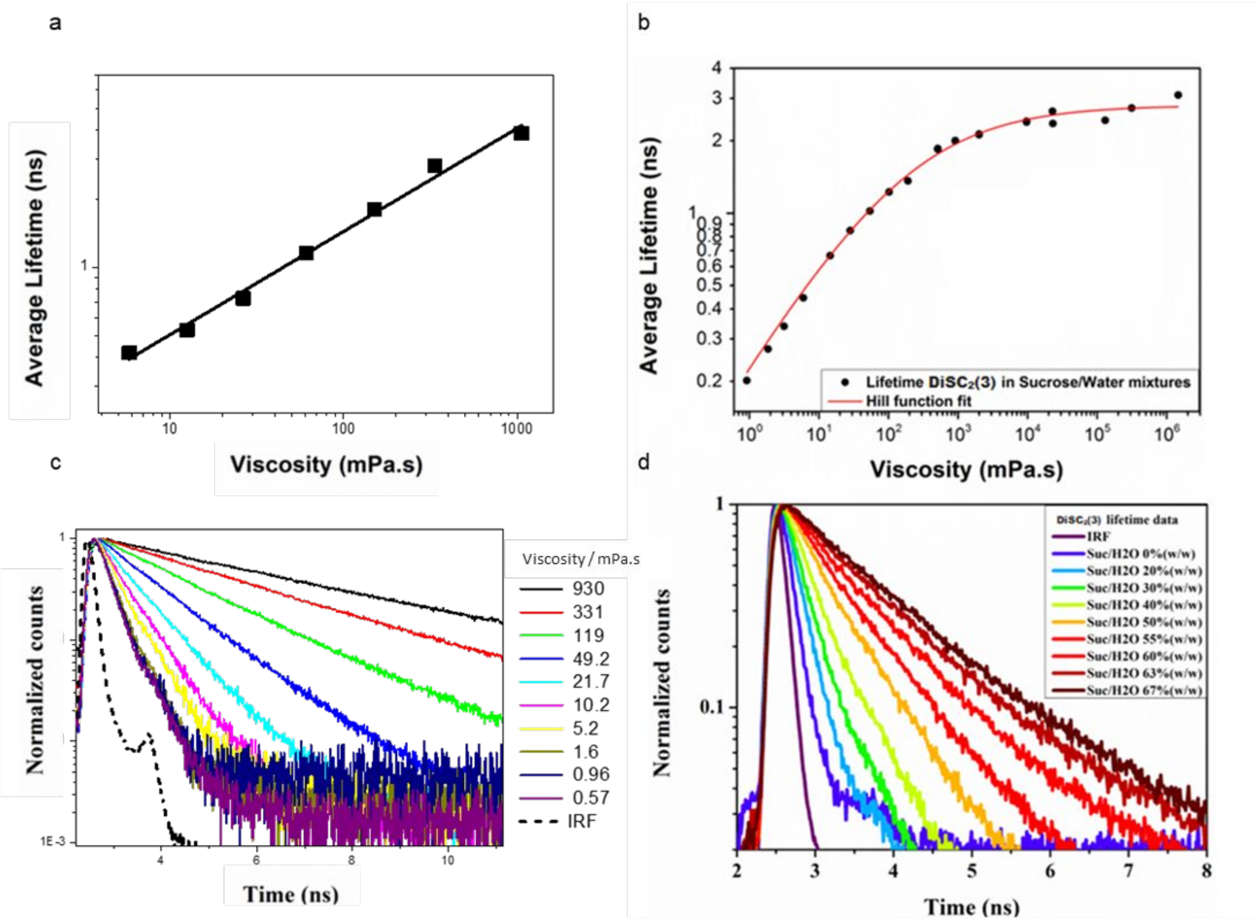

SI Figure 5. Calibration of the molecular rotor lifetimes in solutions of increasing viscosity. a) BODIPY C6++ in methanol/glycerol mixtures[1]. b) $DiSC_2(3)$ in sucrose/water. Decay traces of BODIPY C6++ (c)[1] and $DiSC_2(3)$ (d)[2] in the corresponding solution mixtures.

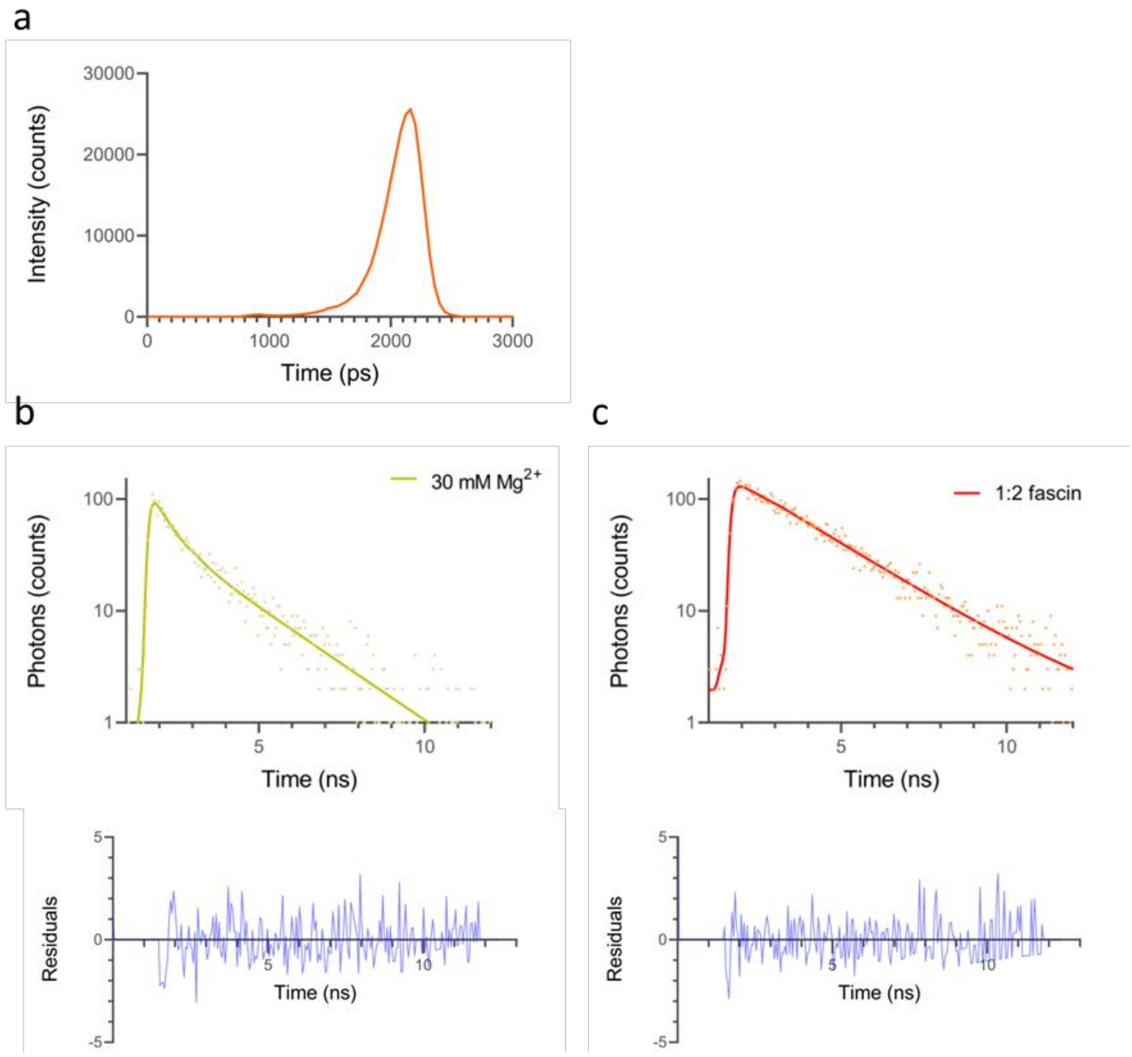

SI Figure 6. a) Distribution histogram of the fascin bundles FLIM image presented in Figure 2f iii. **b)** Decay trace and residuals of 30 mM Mg^{2+} actin bundles presented in Figure 2e. **c)** Decay trace and residuals of fascin bundles presented in Figure 2e. Both residuals are within -5 to +5 (5 Sigma) verifying good fit of the model function to the recorded decay traces.

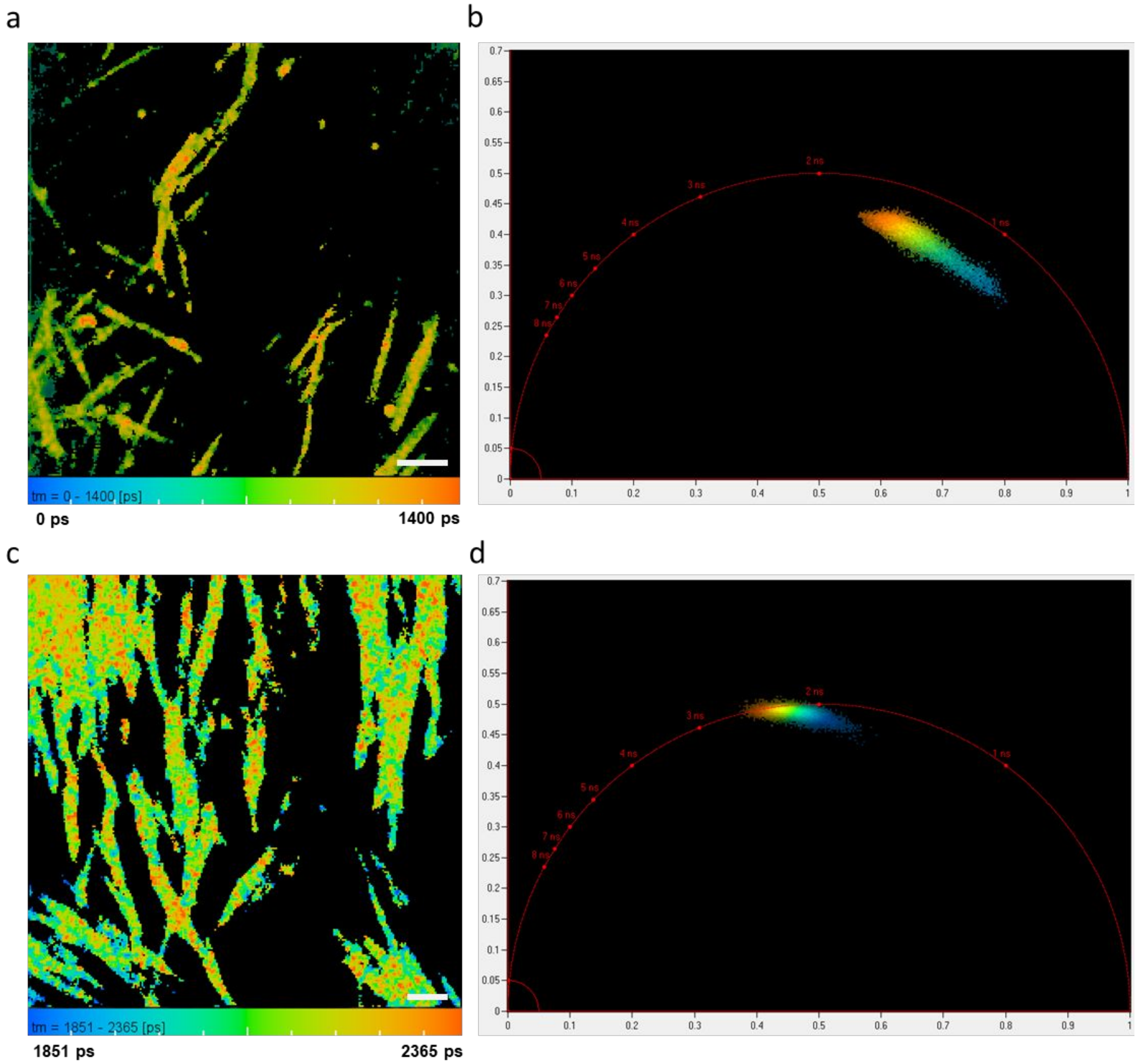

SI Figure 7. FLIM images and the corresponding phasor analysis of various actin bundles, (960 nm excitation, 520-620 nm detection): a) 30 mM Mg^{2+}-mediated actin bundles. **b)** Phasor plot of (a) showing that all pixels produce a phasor distribution inside the universal circle suggesting multiexponential fluorescence decays. **c)** 1:2 actin to fascin bundles. **d)** Phasor plot of (c) where all points are on or close to the universal circle indicating monoexponential fluorescence decays of the rotor. Scalebars are 10 µm.

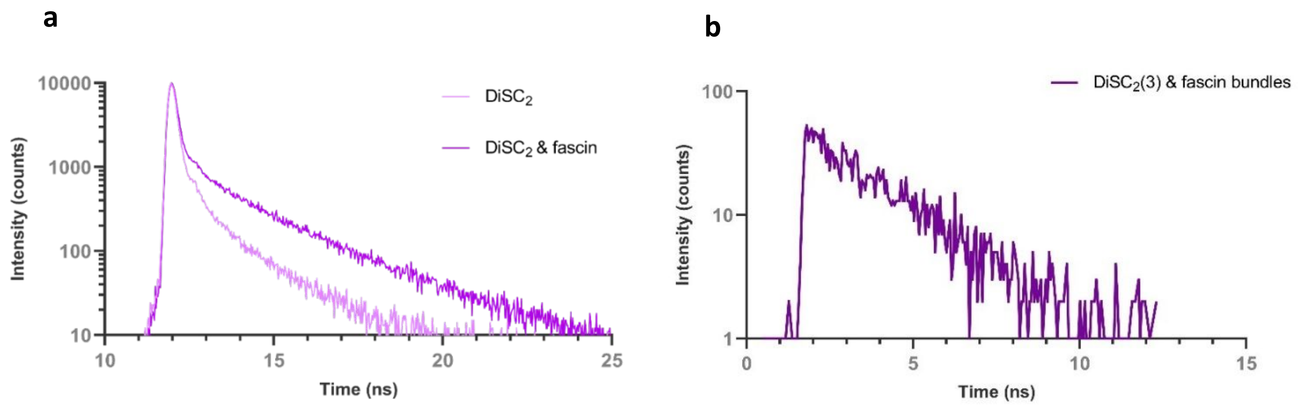

SI Figure 8. **a)** Fluorescence decay traces of $DiSC_2(3)$ and $DiSC_2(3)$ with monomeric fascin, (470 nm excitation, 570 ± 32nm detection). Both conditions present multiexponential decays. **b)** $DiSC_2(3)$ in the presence of fascin bundles follows a monoexponential decay. *The fact that $DiSC_2(3)$ decays differentiate in these two conditions (monomeric fascin and fascin-mediated actin bundles) suggests that there is no strong specific interaction between the rotor and fascin surface and the decay in b) reflects the crowding in fascin-mediated bundles rather than specific binding.*

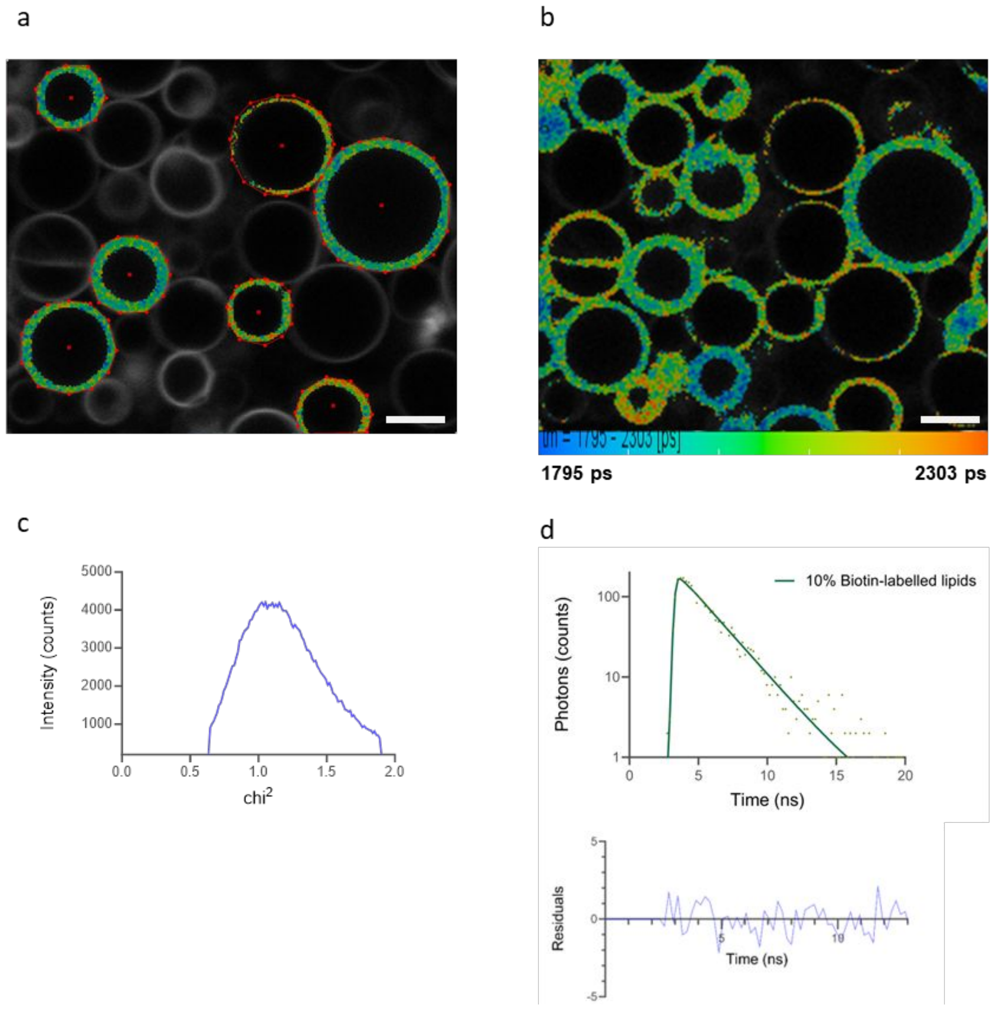

SI Figure 9. **a)** FLIM GUVs images were analysed using the mask selection tool of SPCImage software. Selected analysed regions of individual GUVs can be seen within the red segmentation lines. **b)** Same FLIM image without any selected regions of interest. Both segmented and non-segmented images produce identical results. **c)** Histogram showing the distribution of chi^2 (goodness of fit parameter) following the monoexponential model fit of the image of 10% biotin-labelled GUVs with attached actin formations. **d)** A sample decay trace and residuals of BODIPY++ rotor incorporated into DOPC GUVs with 10% biotin-labelled lipids, from Figure 3d (main text) (488 nm excitation, 500-600 nm detection). Residuals are within -5 to +5 (5 Sigma) suggesting good fit of the model function to the recorded decay traces. Scalebars are 10 µm.

SI Table 1. The mean averaged lifetime values (ps) and their viscosity conversions (cP) of BODIPY++ in GUVs for the rotor alone, as well as the rotor at two attachment conditions: (i) encapsulating streptavidin and (ii) streptavidin and the attached actin formations. The raw data are shown in Figures 3 f, g in the main text. Error (±) values represent standard deviation.

	BODIPY++ alone	streptavidin	attached actin formations
**1% biotin lipids**	1636 ± 143 ps/ 132 ± 0.64 mPa·s	1675 ± 92 ps/ 140 ± 0.2 mPa·s	1700 ± 112 ps/ 145 ± 0.39 mPa·s
**10% biotin lipids**	1943 ± 104 ps/ 193 ± 0.32 mPa·s	2021 ± 84 ps/ 210 ± 0.2 mPa·s	2105 ± 98 ps/ 230 ± 0.3 mPa·s

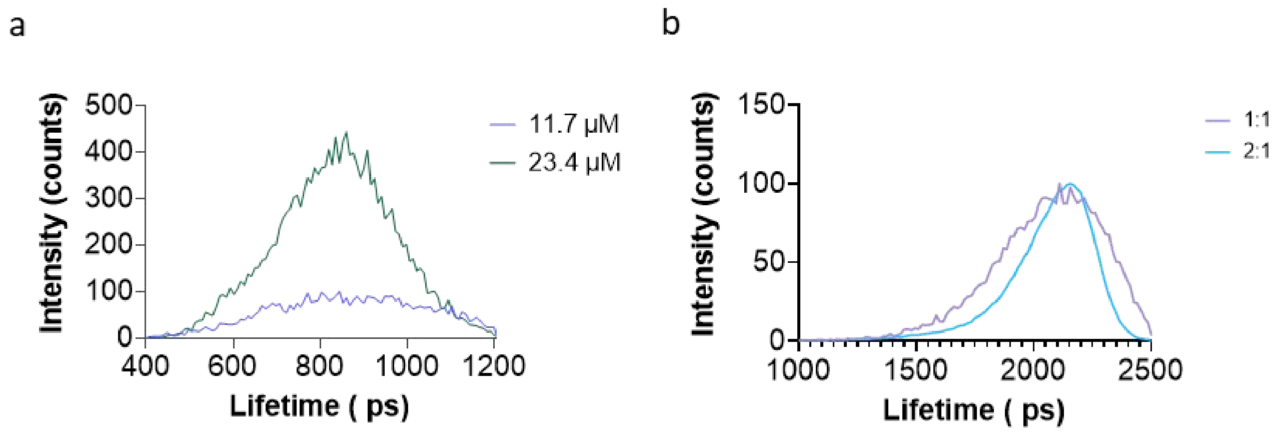

SI Figure 10. a) Lifetime distribution histograms of 30 mM Mg^{2+}-mediated bundles at 11.7 and 23,4 µM. While both histograms are within the same lifetimes range, the 23.4 µM concentration gives higher intensity (stronger recorded signal), revealing in more detail the lifetime variations of the analysed bundles. Thus, we used 23.4 µM $DiSC_2(3)$ in all future imaging experiments. **b)** Lifetime distribution histograms of fascin-mediated bundles with a 1:1 and 1:2 fascin to actin molar ratio. Both histograms present the same range of lifetime values suggesting that even at 1:2 molar ratio, actin bundles are saturated with fascin.

SI References

1 Lopez-Duarte, I., Vu, T. T., Izquierdo, M. A., Bull, J. A. & Kuimova, M. K. A molecular rotor for measuring viscosity in plasma membranes of live cells. *Chem Commun (Camb)* **50**, 5282-5284 (2014). https://doi.org:10.1039/c3cc47530a

2 Hosny, N. A. *et al.* Direct imaging of changes in aerosol particle viscosity upon hydration and chemical aging. *Chem Sci* **7**, 1357-1367 (2016). https://doi.org:10.1039/c5sc02959g
